# Pediatric Mycosis Fungoides and Hydroa Vacciniforme Lymphoproliferative Disorder

**DOI:** 10.3390/cancers18172788

**Published:** 2026-08-27

**Authors:** Christina Cruz, Jennifer Villaseñor-Park, Martin Sangueza, Aaron R. Mangold, Emmilia Hodak

**Affiliations:** 1 Department of Dermatology, Perelman School of Medicine, University of Pennsylvania, Philadelphia, PA 19104, USA; jennifer.villasenor-park@pennmedicine.upenn.edu; 2 Department of Pathology, Hospital Obrero, La Paz P.O. Box 6541, Bolivia; dr.martin.sangueza@gmail.com; 3 Department of Dermatology, Mayo Clinic, Scottsdale, AZ 85259, USA; mangold.aaron@mayo.edu; 4 Division of Oncology, Davidoff Cancer Center, Rabin Medical Center, Beilinson Hospital, Tel Aviv 4941492, Israel; hodake@post.tau.ac.il; 5 Gray Faculty of Medical and Health Sciences, Tel-Aviv University, Tel Aviv 6997801, Israel

**Keywords:** pediatric mycosis fungoides, cutaneous T-cell lymphoma, hypopigmented mycosis fungoides, folliculotropic mycosis fungoides, diagnostic delay, skin-directed therapy, hydroa vacciniforme lymphoproliferative disorder, Epstein–Barr virus

## Abstract

Patients most often present with hypopigmented patches, though folliculotropic disease is also common. Prognosis is usually excellent. Most patients are diagnosed with early-stage disease, and less than 5% of cases progress to late-stage disease. Pediatric MF can be difficult to recognize. It often mimics more common benign inflammatory and pigmentary dermatoses. Furthermore, clinicians may be hesitant to perform skin biopsies in younger patients, contributing to delays in diagnosis. Evaluation and treatment guidelines largely follow those of adult MF, with special considerations regarding age and expected lifetime exposure to therapy. Adaptations include limiting unnecessary ionizing radiation, relying more heavily on ultrasound as an imaging modality, and balancing disease control with the long-term safety of treatments, including topical corticosteroids and mutagenic therapies such as PUVA, mechlorethamine gel, and radiation. Despite favorable outcomes, the chronic nature of MF can lead to substantial psychosocial burden on both the patients and their families. Caregivers must navigate frequent visits, uncertainty about recurrence, and concerns about long-term adverse effects. Management should therefore incorporate developmentally appropriate communication, psychosocial support, and long-term follow-up. This chapter reviews the epidemiology, clinical and pathologic features, diagnostic approach, staging, and treatment of pediatric MF. It highlights the distinctions between pediatric and adult disease, recently developed recommendations, and the importance of individualized, family-centered care. Because the clinician evaluating a child with an atypical or persistent eruption must distinguish pediatric MF from other rare cutaneous lymphoproliferative disorders, this review also discusses hydroa vacciniforme lymphoproliferative disorder (HV-LPD). Although HV-LPD is a distinct Epstein–Barr virus-–associated entity rather than a variant of MF, it is included here because it is one of the few primary cutaneous lymphoproliferative disorders of childhood. It therefore shares the diagnostic challenges of mimicking benign dermatoses and requiring careful clinicopathologic and immunophenotypic correlation, and represents an important consideration in the pediatric differential diagnosis.

## Simple Summary

Pediatric mycosis fungoides is a rare cutaneous T-cell lymphoma that most often presents with hypopigmented disease. Although prognosis is usually excellent, diagnosis may be delayed for several reasons. Firstly, skin findings frequently resemble benign dermatoses. Additionally, biopsies tend to be avoided in pediatric patients. Evaluation and treatment largely follow adult MF guidelines but should take into account the age of the patient, i.e., minimize ionizing radiation and account for the long-term safety of therapy. Management is usually centered on skin-directed treatments, including topical corticosteroids and phototherapy. Psychosocial support and long-term follow-up are also critical for the emotional well-being of the patient and their caregivers. Hydroa vacciniforme lymphoproliferative disorder is an important Epstein–Barr virus-associated condition in the pediatric differential diagnosis and may range from skin-limited disease to progressive systemic involvement.

## 1. Introduction

Pediatric mycosis fungoides (MF) is a form of cutaneous T-cell lymphoma (CTCL) with unique clinical features and considerations compared to MF seen in adults. It is quite rare, with an estimated incidence of 0.1–0.3 cases per million person-years [1,2,3,4,5,6]. Patients most often present with hypopigmented patches, though folliculotropic disease is also common [2,3,7,8,9,10,11,12]. Prognosis is usually excellent. Most patients are diagnosed with early-stage disease, and less than 5% of cases progress to late-stage disease [1,2].

Pediatric MF can be difficult to recognize. It often mimics more common benign inflammatory and pigmentary dermatoses. Furthermore, clinicians may be hesitant to perform skin biopsies in younger patients, contributing to delays in diagnosis [1,4,13]. Evaluation and treatment guidelines largely follow those of adult MF, with special considerations regarding age and expected lifetime exposure to therapy. Adaptations include limiting unnecessary ionizing radiation, relying more heavily on ultrasound as an imaging modality, and balancing disease control with the long-term safety of treatments, including topical corticosteroids and mutagenic therapies such as PUVA, mechlorethamine gel, and radiation [4,14].

Despite favorable outcomes, the chronic nature of MF can lead to substantial psychosocial burden on both the patients and their families. Caregivers must navigate frequent visits, uncertainty about recurrence, and concerns about long-term adverse effects [2,3,15,16,17,18]. Management should therefore incorporate developmentally appropriate communication, psychosocial support, and long-term follow-up.

This chapter reviews the epidemiology, clinical and pathologic features, diagnostic approach, staging, and treatment of pediatric MF. It highlights the distinctions between pediatric and adult disease, recently developed recommendations, and the importance of individualized, family-centered care.

Related pediatric cutaneous lymphoproliferative disorders and their distinguishing clinical and pathologic features are also discussed. In particular, this review includes hydroa vacciniforme lymphoproliferative disorder (HV-LPD). HV-LPD is not a subtype or variant of MF. It is a biologically distinct, Epstein–Barr virus–driven T-/NK-cell lymphoproliferative disorder with its own clinical spectrum, histopathology, and prognosis. It is discussed alongside pediatric MF for three reasons. First, both are rare primary cutaneous lymphoproliferative disorders that can affect children, a population in which such diagnoses are seldom considered. Second, both frequently mimic benign inflammatory dermatoses and pose similar diagnostic challenges for clinicians. Correct diagnosis depends on integrated clinicopathologic, immunophenotypic, and—in the case of HV-LPD—virologic correlation. Third, both should be considered in the differential diagnosis of a child with persistent or refractory skin lesions. Grouping them therefore reflects shared diagnostic approaches and considerations rather than any shared pathogenesis.

## 2. Epidemiology and Clinical Characteristics of Pediatric MF

### 2.1. Epidemiology

Mycosis fungoides (MF) is a rare cutaneous T-cell lymphoma, representing approximately 1–2% of non-Hodgkin lymphomas. It is the most common primary cutaneous lymphoma and is typically diagnosed in adults aged ≥ 50 years, with an incidence of about 6 cases per million [19]. In children and adolescents, MF is uncommon, with an estimated incidence of 0.1–0.3 cases per million person years among individuals aged ≤ 19 years and varies widely by region: it accounts for approximately 0–3% of all CTCL cases in North America and Europe, with higher reported rates of 5.8–16% in the Middle East, 18% in South America, and 16–25% in the Far East [1,4,5,6]. This may represent either true variation in frequency or differing indices of suspicion. Most pediatric patients are diagnosed with MF between the ages of 10 and 15 years of age, with a slight male predominance [1]. This sex disparity is more pronounced in adult MF, where the male: female ratio is closer to 2:1. Racial disparities also differ between pediatric and adult MF. Whereas the incidence of adult MF is highest in Black populations, the incidence of MF under 30 is highest in Asians, Pacific Islanders, and Black patients [20].

### 2.2. Clinicopathologic Characteristics

The clinical presentation of MF varies between children and adults. Key differences are summarized in Table 1. In adults, the classical Alibert-Bazin MF variant is most common, whereas children more frequently present with the hypopigmented variant [7,8]. This hypopigmented form appears as pale or achromic patches and comprises 48–56% of pediatric cutaneous T-cell lymphoma cases [1,2,3]. In pediatric MF, the predominant immunophenotype is CD8+, found in up to 39% of cases, which aligns with the high prevalence of hypopigmented MF [1,21,22,23,24]. Other cytotoxic markers, such as CD56 and TIA-1, are also more frequently observed in children [23,25].

The folliculotropic variant (FMF) is the second most common MF variant among children, representing up to 49% of cases across diverse ethnic backgrounds [2,3,9,10,11,12]. FMF may present subtly, with thin scaly patches, follicular prominence, keratosis pilaris-like features, or acneiform lesions, often affecting the trunk and extremities and sometimes accompanied by variable alopecia [2,26]. Histologically, early FMF shows features of folliculotropism with or without mucin and sparse infiltrates of CD4+ atypical T-cells confined to the superficial dermis [26]. In contrast, advanced stages of FMF commonly present with thick plaques and/or tumors with a dense follicular and perifollicular infiltrate of atypical cells that often extend deeper in the dermis in a nodular or diffuse pattern [26].

Pediatric MF also frequently presents with mixed morphologic types, such as hypopigmented patches alongside lesions typical of FMF [1,2,4]. Classical MF lesions are also seen in children, while rarer variants—including pagetoid reticulosis, granulomatous slack skin, and poikilodermatous MF—make up less than 5% of pediatric MF cases [1,27].

Due to its rarity, clinical presentations often resemble benign dermatoses, and clinicians’ hesitance to biopsy young patients, there is often a significant delay in diagnosis, typically ranging from 1 to 5 years, with some reports of delays of 14–24 years [23,28].

### 2.3. Clinical Course

The prognosis for pediatric patients with mycosis fungoides (MF) is exceedingly favorable, with a reported 10-year disease-specific survival rate of approximately 95% [1]. This likely reflects the frequent early-stage presentation observed in this population, as the majority of patients exhibit limited cutaneous involvement without evidence of lymph node, hematologic, or visceral disease [1,13,20,29]. Further, 85–97% of patients present with stage IA–IIA disease. Disease progression beyond early-stage disease (Stage IA–IIA) or large cell transformation occurs in fewer than 5% of patients [1,2].

## 3. Molecular Pathogenesis and Emerging Biomarkers

Pediatric-specific molecular data in MF remain sparse, and most understanding of its biology is extrapolated from adult data. Because pediatric MF shares core histologic and phenotypic features with adult early-stage disease, a brief overview of its mechanisms provides useful context and highlights directions of relevance to children.

### 3.1. Genomic Landscape

A defining feature of MF is the predominance of copy-number and structural variation over point mutations, and mutational burden increases sharply with progression. For example, TP53 is deleted in roughly 7% of MF cases and 87% of SS cases [30,31]. Pediatric data are limited but suggest that pediatric MF is not simply a younger version of the adult genome. It carries a lower mutation burden, which is consistent with its indolent behavior [32]. Notably, a recent study identified recurrent in-frame tyrosine kinase gene fusions, with JAK2 fusions in 64% (7/11) of pediatric MF cases and a TYK2 fusion in another. Not only are these alterations distinct from the JAK/STAT activation of adult disease, but they may be targetable for treatment [33].

### 3.2. Epigenetic and Non-Coding RNAs

Epigenetic remodeling is common in CTCL and includes global DNA hypomethylation, focal tumor-suppressor promoter hypermethylation, and overexpression of EZH2 [34,35]. MicroRNA patterns can differentiate MF from benign dermatoses, and a validated three-microRNA classifier can predict progression to advanced-stage disease [36,37]. It is not yet known whether these findings apply similarly to pediatric disease.

### 3.3. Telomere Biology and TERRA

Aggressive subtypes show short telomeres with retained telomerase activity, which promotes tumor-cell survival through functions beyond telomere lengthening [38]. CTCL cells can re-express the catalytic telomerase subunit hTERT. Chebly et al. showed that this may occur through epigenetic changes, with marked hypermethylation of the TERT hypermethylated oncogenic region (THOR) in CTCL cells compared with healthy CD4+ and CD34+ cells. Thus, THOR hypermethylation may serve as a characteristic feature and potential biomarker of CTCL [39]. Treatment with HDAC inhibitors or 5-azacytidine reduced hTERT expression without changing THOR methylation, suggesting that these therapies act through indirect mechanisms [39]. The telomeric long non-coding RNA TERRA is also altered in CTCL, with decreased TERRA 16p and increased TERRA 11q expression reported during lymphomagenesis [40]. Whether these mechanisms differ between pediatric and adult CTCL remains unknown.

### 3.4. Immune Escape and Tumor Microenvironment

As CTCL progresses, the immune environment shifts from a Th1- to a Th2-dominant response, with chemokine signaling and Staphylococcus aureus superantigens promoting immune escape and STAT3 activation [41]. Checkpoint expression (PD-1, PD-L1, and ICOS) also increases with progression and large-cell transformation and is associated with clinical outcomes [42,43]. Additionally, M2 macrophages, cancer-associated fibroblasts, and CXCL13-mediated recruitment of B cells help create a tumor-supportive microenvironment [44].

### 3.5. Metabolic Reprogramming

CTCL cells alter their metabolism to support proliferation and protect against oxidative stress [45]. Malignant T-cells show a Warburg-type shift, increasingly rely on glycolysis, with higher expression of HIF-1α and glycolytic enzymes in more advanced disease. Inhibition of mTOR with rapamycin has been shown to reduce glycolysis and tumor growth [46,47]. CTCL cells may also depend on glutamine metabolism. In addition, an imbalance in antioxidant proteins, with high SOD2 and low PRDX2 expression, leaves these cells especially vulnerable to further increases in reactive oxygen species. This may help explain why romidepsin-induced cell death is dependent on oxidative stress [48]. Whether these metabolic features are also present in indolent pediatric MF remains unknown.

### 3.6. Prognostic Biomarkers

Several molecular biomarkers may help predict disease behavior in CTCL. These include specific T-cell receptor (TCR) clonotypes, which are associated with advanced-stage disease, and transcriptional classifiers, which can both distinguish MF from other conditions and identify patients at higher risk of progression [49,50]. Among circulating markers, plasma CXCL13 differentiated SS from MF and inflammatory dermatoses with an AUC of 93% and was significantly lower in early-stage compared to advanced-stage MF [51]. Given the non-clonal, low-mutation-burden phenotype of pediatric MF, one cannot assume that these findings apply to children. Dedicated studies in pediatric disease are needed [32,33].

## 4. Diagnosis, Staging, and Treatment of Pediatric Early-Stage MF

**Figure 1:**
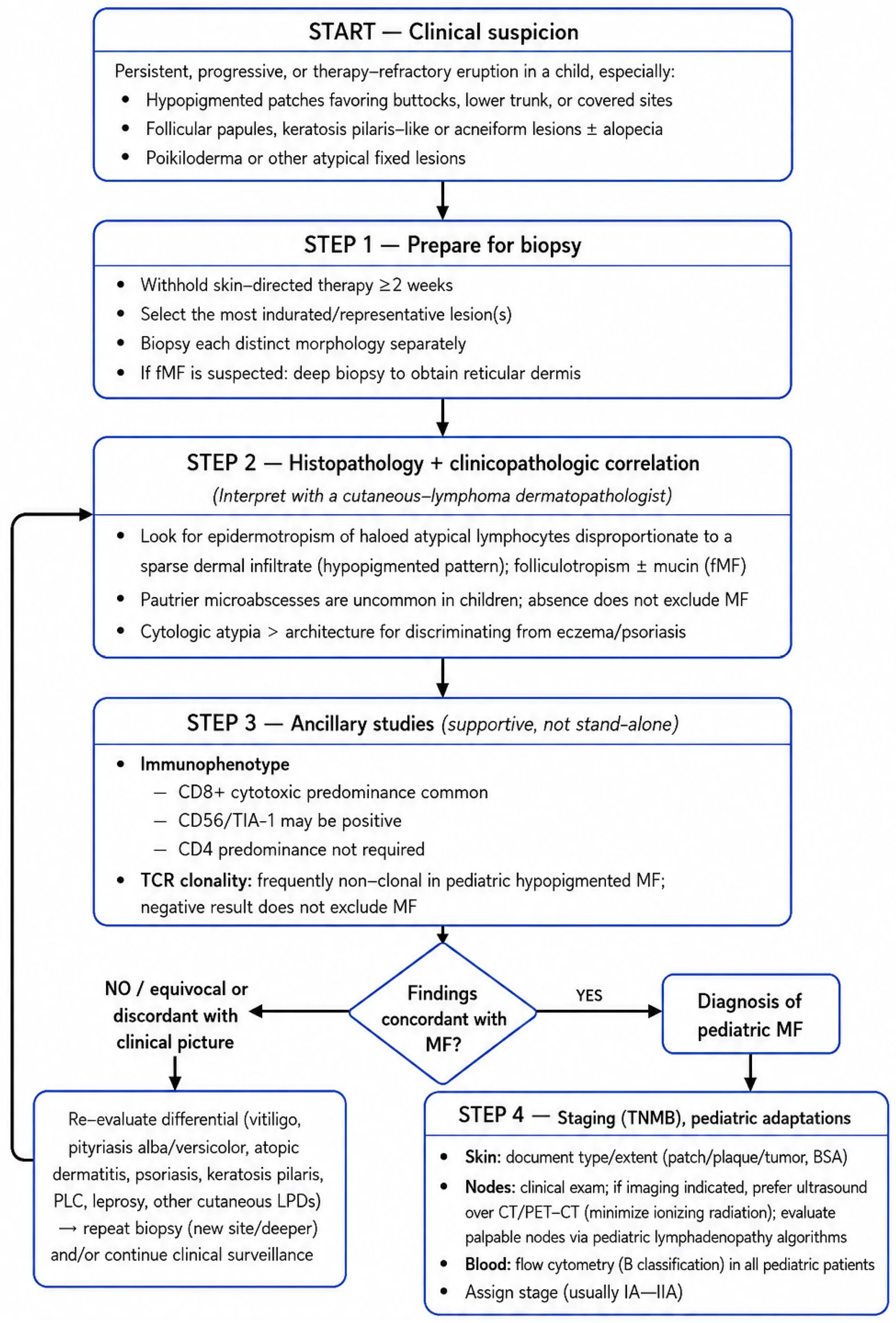
Diagnostic algorithm for pediatric mycosis fungoides.

In recognition of the unique nature of pediatric MF and safety considerations specific to children and adolescents, staging and treatment Delphi-based recommendations tailored to early-stage pediatric MF have recently been developed by a joint effort between the International Society for Cutaneous Lymphomas (ISCL), the United States Cutaneous Lymphoma Consortium (USCLC), and the European Organization for Research and Treatment of Cancer—Cutaneous Lymphoma Tumor Group (EORTC-CLTG) [4,14]. A proposed diagnostic algorithm can be found in Figure 1.

### 4.1. Diagnostic Approach and Differential Diagnosis

Guidelines for diagnosing MF generally support interpreting the clinical picture, histopathology, ancillary immunophenotyping, and T-cell clonality studies together. This framework equally applies to diagnosing MF in children, though there are a few special considerations in this population [52]. What differs in the pediatric setting is the threshold for suspecting the disease, the histologic and phenotypic patterns most often encountered, and the set of benign conditions that must be excluded, which are referenced in Table 2.

#### 4.1.1. When to Biopsy

Clinical features alone cannot diagnose MF—histopathologic confirmation is required [20]. In children, the initial barrier to obtaining a biopsy is recognizing the need for one. Because pediatric MF is relatively rare and often presents with benign-appearing lesions, it may not initially be considered in the differential diagnosis. A second barrier comes in the form of hesitation to biopsy a young patient due to the psychological trauma that may accompany the procedure. Biopsy should be pursued for a lesion that is persistent, progressive, or refractory to appropriate therapy. It should also be considered when an eruption favors sites on the body that are not exposed to the sun, such as the buttocks, lower trunk, and proximal limbs. Hypopigmented lesions over the buttocks can be particularly suggestive of MF in children [20,52,55]. Importantly, a prior biopsy showing a benign pattern does not exclude MF [20].

Given the disease distribution and common variants in pediatric MF, several preparatory measures are especially relevant. Because treatment may attenuate the epidermotropic infiltrate, skin-directed topical therapies such as topical corticosteroids should be withheld for at least two weeks before biopsy. It is also important to note that in the folliculotropic variant, which is very common in children, biopsy specimens must reach the deep dermis in order to properly evaluate the tissue. If FMF is suspected, a superficial shave should be avoided.

#### 4.1.2. Clinicopathologic Correlation

Histopathologic findings in MF must always be interpreted within the clinical context, ideally by a dermatopathologist with expertise in cutaneous lymphoma. When biopsy findings are nondiagnostic or discordant with the clinical presentation, repeat biopsy is recommended [4,52]. Several histopathologic features of pediatric MF differ from those seen in adults. Pautrier microabscesses, although characteristic of adult MF, are distinctly uncommon in pediatric cases, so their absence should not weigh strongly against the diagnosis [32]. Instead, particular attention should be paid to the classic pattern of hypopigmented MF, which is characterized by prominent epidermotropism of haloed atypical lymphocytes that is disproportionate to a relatively mild dermal infiltrate [55]. Across pediatric MF variants, cytologic atypia appears to carry greater discriminatory value than overall tissue architecture when distinguishing MF from inflammatory mimickers such as atopic dermatitis [56].

#### 4.1.3. The Need for Multiple Biopsies

It is common for children to present with more than one morphology. For example, hypopigmented patches and keratosis pilaris-like follicular lesions are frequently seen in the same pediatric patient. In these cases, each morphology should be biopsied separately, since folliculotropism and large-cell transformation influence therapy [52]. The benefit of serial biopsy over time must be weighed against the psychosocial cost of uncertainty for the child and family, as well as the discomfort and psychological burden of the biopsies themselves for the children.

#### 4.1.4. Ancillary Studies: Clonality and Immunophenotyping

TCR clonality studies and immunophenotyping can provide useful support when interpreting a clinical picture and establishing a diagnosis. Several pediatric-specific considerations are important. Because pediatric MF is predominantly hypopigmented, it frequently demonstrates a CD8+ cytotoxic phenotype, and a CD4-predominant infiltrate should not be expected [20]. Accordingly, the absence of CD4 predominance should not be interpreted as evidence against the diagnosis. CD56 and TIA-1 are also reported more commonly in pediatric than adult MF [32]. Similarly, pediatric hypopigmented MF is frequently non-clonal, making a negative clonality study particularly unreliable in this population [32].

### 4.2. Staging

Staging of MF is based on the TNMB (tumor, node, metastasis, blood) classification, ranging from stage IA to IVB [57,58]. While the overall diagnostic and staging approach in pediatric MF largely parallels that used in adults, several important differences should be noted. Most importantly, when imaging is indicated in early-stage pediatric MF, assessment of lymph nodes (LNs) should rely primarily on ultrasound rather than computed tomography (CT) with contrast or integrated positron emission tomography (PET-CT), which are used in adults [4]. This recommendation reflects the rarity of systemic involvement in early presentations of MF in general [59] and in pediatric MF in particular. Furthermore, children and adolescents are more susceptible than adults to the harmful effects of ionizing radiation, and their longer life expectancy increases the risk of radiation-induced secondary malignancies [60,61,62]. Clinicians should also recognize that palpable LNs are relatively common in children due to the higher prevalence of infectious diseases [63]. Therefore, when lymphadenopathy is detected, it should be evaluated according to established pediatric algorithms for the assessment of enlarged LNs, balancing the need to avoid unnecessary LN biopsies while ensuring timely diagnosis in the rare instances of underlying malignancy [64].

Finally, flow cytometry (FC) has become the standard method for evaluating peripheral blood involvement in MF and forms the basis for the B classification (B0–B2), whereas manual Sézary cell counting based solely on morphology is no longer required. Some guidelines recommend FC analysis in all stages of adult MF [57,65], whereas others consider it optional in early-stage disease [66] or optional specifically in patients with T1 disease [58]. Given the sparse data regarding FC analysis in pediatric MF, the current recommendation suggests that FC-based assessment of blood involvement should be performed in all pediatric patients until further studies clarify its clinical utility [4,67].

### 4.3. Treatment

Treatment of pediatric MF is based primarily on the standard practice in adults [58,65,66,68]. However, some of the therapeutic modalities available for adult MF are used off-label or relatively contraindicated in the pediatric age group. In selecting the appropriate therapy, both disease-specific parameters and patient-related factors should be taken into account. The recent ISCL, EORTC-CLTG, and USCLC recommendations for the treatment of early-stage pediatric MF are outlined in Table 3 [14]. The basis for these recommendations is summarized as follows: The most commonly administered therapies are topical corticosteroid (TCS) and narrow-band UVB (NBUVB) [1].

Published data on TCS indicate that, similarly to adults, the overall response rate (ORR) in pediatric MF might be high, but complete response (CR) rates are variable (10–33%) [13]. A common approach is to begin with a high-potency steroid for a limited course to achieve prompt disease control, and then subsequently reduce to once- or twice-weekly maintenance when needed [14]. Topical corticosteroids are generally well-tolerated in children and have an overall favorable safety profile when used appropriately [69].

Yet, given the potential for a higher degree of systemic absorption of TCS compared to adults, TCS should be used mainly for limited surface areas (stage IA) [70].

NB-UVB appears to be an effective therapy for pediatric MF, and similar to adults, it is suitable for patch/flat plaque [57,66]. Hypopigmented MF is known for its high rates of response to NB-UVB [20,71,72], but this is not the case for FMF (CR in FMF vs. non-FMF, 29% vs. 63%) [2]. Expert opinion is that it is a safe treatment in children [73]. However, they are mainly based on short-term follow-up, especially in pediatric psoriasis cases, while studies are lacking for MF. Furthermore, long-term safety data are strikingly lacking [74,75].

Similar to adult MF, UVA-based therapy is reserved for pediatric MF with thicker plaques or FMF, as UVA penetrates more deeply into the dermis than UVB [57,76,77]. In general, due to safety issues, the use of oral psoralen–ultraviolet A (systemic PUVA) in children is considered off-label therapy, should be used with caution, and is relatively contraindicated in children younger than 12 years of age [73]. Therefore, other UVA-based therapies with a better safety profile and fewer adherence issues should be preferred over systemic PUVA, if possible. This includes: topical PUVA—so far has been sporadically reported to be an effective therapy for early FMF [2,9,78]; UVA1—was found to be an effective therapy in a few pediatric MF patients [79,80]; combined UVA and NB-UVB—reports suggested encouraging results in both adult and pediatric patients with early MF [2,9,72,81]. A more recent retrospective analysis [39 with FMF (14 children) and 12 non-FMF (2 children)] showed that this modality is a good alternative to systemic PUVA for adult and pediatric early FMF or non-FMF, including cases that were refractory to NB-UVB [82]. Importantly, although as opposed to systemic PUVA, these UVA-based alternatives to systemic PUVA have not been linked with increased risk for cutaneous non-melanoma skin cancer; however, due to the limited data, long-term patient surveillance for side effects should be continued [14].

Topical chlormethine (mechlorethamine) is an established first-line option for patch- and plaque-stage MF in adults, but it is not currently approved for use in patients under 18 years of age. Evidence supporting its use in pediatric MF remains limited [1], with most published experience derived from older compounded formulations rather than currently available preparations [2,21,83].

It may be less suitable for children due to its known high rates of cutaneous side effects, which can affect adherence [21], and the lack of data regarding systemic absorption in children [84]. Therefore, given the limited experience and safety concerns, it should be used with caution as a second-line therapy and mainly for limited skin involvement.

Systemic retinoids and interferon alpha, the most common systemic treatments used in early- stage adult MF, are rarely employed in pediatric patients [1] as the vast majority respond well to skin-directed therapies.

When systemic treatment is necessary, retinoids are typically favored over interferon alfa, reflecting the broader pediatric experience with retinoids, particularly RAR-targeting agents used in inflammatory disorders, as well as concerns regarding interferon alfa toxicity [14]. In contrast, pediatric experience with bexarotene remains very limited, which has restricted its use in this population [85]. Methotrexate is another potential option, used second-line for refractory or recurrent disease, supported largely by its extensive use and familiarity in pediatric practice.

## 5. Hydroa Vacciniforme Lymphoproliferative Disorder

### 5.1. Definition and Epidemiology

Hydroa vacciniforme lymphoproliferative disorder (HV-LPD) is a cutaneous manifestation of chronic active Epstein–Barr virus (EBV) infection that primarily affects children and young adults [86,87,88,89,90]. It encompasses a broad clinical spectrum, ranging from a self-limited photodermatosis, termed classic HV-LPD, to a more aggressive systemic disorder with extensive skin lesions and multiorgan involvement [91,92]. The systemic form was previously referred to as severe hydroa vacciniforme or hydroa vacciniforme-like lymphoma [93,94,95].

HV-LPD has been described mainly in Asia, Mexico, and Central and South America and is rare in Caucasian populations. Patients typically present during childhood or adolescence, with a slight male predominance [91,92,96,97,98]. EBV infection is a key factor in the development of HV-LPD, although genetic, ethnic, and environmental factors may contribute to its clinical behavior and geographic distribution [99].

### 5.2. Clinical Features

Classic HV-LPD typically presents with recurrent papulovesicular lesions on areas of the skin exposed to sun, such as the face and extremities. The lesions evolve into blisters and ulcers and heal with characteristic varioliform scars. Skin lesions frequently show seasonal variation, with recurrences occurring more often during the spring and summer. Some cases resolve during adolescence, whereas others have a more protracted or relapsing course [91,96,100,101].

In systemic HV-LPD, lesions are not clearly induced or exacerbated by sun exposure and may involve both sun-exposed and non-sun-exposed areas. As the disease progresses, patients may develop severe and extensive skin lesions associated with fever, weight loss, lymphadenopathy, and hepatosplenomegaly [91,92,94,97]. Deep ulceration, necrosis, and prominent facial, periorbital, or lip edema may also occur [101,102]. Some patients have concomitant hypersensitivity to mosquito bites [103].

### 5.3. Histopathology and Immunophenotype

Histologically, the skin may show epidermal reticular degeneration or spongiosis with vesiculation, necrosis, and, in advanced cases, ulceration. A perivascular and periadnexal infiltrate of atypical small- to medium-sized lymphocytes is present within the dermis and may extend into the subcutaneous tissue. Angiotropism, angiodestruction, and tissue necrosis may also be present [91,92,96,104,105].

The atypical lymphocytes most commonly have a CD8-positive cytotoxic T-cell phenotype with expression of TIA-1, granzyme B, or perforin [94,101,105]. Less commonly, the lymphocytes have a CD4-positive, CD4-negative/CD8-negative, or natural killer cell phenotype. Cases with a natural killer cell phenotype may express CD56 and show prominent involvement of the subcutaneous tissue, mimicking subcutaneous panniculitis-like T-cell lymphoma [91,98,106].

In situ hybridization demonstrates EBV-encoded small RNA within variable numbers of atypical lymphocytes [91,98]. Most cases show clonal rearrangements of the T-cell receptor genes [91,92,96,97,99]. However, T-cell clonality, the number of EBV-positive cells, and the density of the lymphocytic infiltrate do not reliably correlate with the clinical course [92,103].

### 5.4. Diagnosis and Differential Diagnosis

The diagnosis of HV-LPD requires correlation of the clinical presentation with the histopathologic and immunophenotypic findings because morphology and immunophenotype alone may resemble other T-cell lymphomas [91,105]. The differential diagnosis includes extranodal NK/T-cell lymphoma, subcutaneous panniculitis-like T-cell lymphoma, and primary cutaneous gamma-delta T-cell lymphoma [90,91,92,96].

### 5.5. Clinical Course and Treatment

The clinical course of HV-LPD is variable. Classic HV-LPD often follows a chronic, relapsing course and may resolve by adolescence or adulthood, particularly in Caucasian patients [99]. In contrast, systemic HV-LPD may be associated with progressive extracutaneous involvement and the development of systemic lymphoma [92].

There are currently no standardized treatment guidelines for HV-LPD. In patients with classic, skin-limited disease, a conservative approach with photoprotection, topical corticosteroids, and close clinical follow-up may be used [94,96,101]. Systemic disease has been treated with immunomodulatory therapies, including thalidomide or intravenous immunoglobulin, although responses are variable [107,108]. Hematopoietic stem cell transplantation may be considered in patients with severe systemic disease or lymphoma [109].

## 6. Conclusions

Pediatric mycosis fungoides is a rare cutaneous T-cell lymphoma with distinct characteristics and presentation. Hypopigmented and folliculotropic variants are most common, and resemblance to more common benign dermatoses often leads to diagnostic delay. Despite these challenges, prognosis is excellent, with most patients presenting with early-stage disease and rarely progressing beyond that, even long-term.

Given the young patient population, thoughtful adaptation of both diagnostic and therapeutic approaches is required. Management should balance effective disease control with the potential cumulative risks of imaging and treatment. Limiting ionizing radiation, using skin-directed therapies when possible, and taking into account long-term adverse effects are central to care. Providers must also consider the emotional and social effects of chronic disease on patients and their families.

Despite recent progress, several important gaps remain. First, the molecular biology of pediatric MF is largely unexplored. Most mechanistic understanding is extrapolated from adult disease. However, emerging pediatric-specific findings—such as recurrent JAK2 and TYK2 tyrosine kinase fusions and a frequently non-clonal, CD8+, low-mutation-burden profile—suggest that childhood disease may be biologically distinct. Dedicated studies on genomics, epigenetics, immunophenotyping, biomarkers, and microenvironment in pediatric cohorts are needed to understand this distinct disease and identify potential targets.

Second, due to the rarity of this disease, it is difficult to generate high-quality evidence from any single center. Multi-center studies and international registries are essential to understand disease mechanisms, standardize diagnosis and treatment strategies, and define long-term outcomes across variants.

Third, data on long-term therapeutic outcomes are strikingly limited. Cumulative efficacy and safety of skin-directed therapies, phototherapy, and mutagenic modalities over the decades of life expectancy typical of these patients remain poorly characterized. Longitudinal follow-up is required to quantify secondary malignancy risk, relapse patterns, and quality-of-life trajectories.

Although HV-LPD is a biologically distinct entity, it is included here because it shares the diagnostic space of pediatric cutaneous lymphoproliferative disease, mimicking benign dermatoses and requiring integrated clinicopathologic, immunophenotypic, and virologic correlation.

Ultimately, timely diagnosis and individualized, multidisciplinary, and family-centered care are essential to optimizing both clinical outcomes and quality of life for children and adolescents with cutaneous lymphoproliferative disease. Addressing these molecular, collaborative, and long-term outcome gaps will be central to advancing the field.

## Figures and Tables

**Table 1:** Key differences between MF in pediatric and adult populations.

Feature	Adult MF	Pediatric MF
Typical age and incidence	≥50 yr; ~6 per million	≤19 yr; 0.1–0.3 per million person-years; usually 10–15 yr
Sex ratio	Male:female ~2:1	Slight male predominance
Most common variant	Classic Alibert-Bazin	Hypopigmented (48–56%)
Second most common	Folliculotropic (15%)	Folliculotropic (up to 49%)
Predominant immunophenotype	CD4+	CD8+ cytotoxic (up to ~39%); CD56/TIA-1 more frequent
TCR clonality	Frequently clonal	Frequently non-clonal (esp. hypopigmented)
Pautrier microabscesses	Characteristic	Distinctly uncommon
Stage at presentation	More variable	85–97% early-stage (IA–IIA)
Prognosis	Stage-dependent; poorer in advanced	Excellent; ~95% 10-yr disease-specific survival; <5% progress
Nodal imaging	CT/PET-CT	Ultrasound preferred
Blood assessment	Flow cytometry (variably required by stage)	Flow cytometry in all patients
Treatment emphasis	Full adult armamentarium	Skin-directed (TCS, NBUVB); caution with PUVA/chlormethine (relatively contraindicated <12 yr)

**Table 2:** Differential diagnosis organized by clinical variant and distinguishing clues that favor a diagnosis of MF [53,54].

MF Variant	Clinical Appearance	Principal Mimickers	Distinguishing Clues Favoring MF
Hypopigmented (most common)	Pale/achromic patches, favoring buttocks, lower trunk, covered sites	Vitiligo, pityriasis alba, atopic dermatitis, post-inflammatory hypopigmentation, progressive macular hypomelanosis, pityriasis versicolor, indeterminate/tuberculoid leprosy	Persistent/progressive despite therapy; non-sun-exposed distribution; intense epidermotropism by haloed atypical CD8+ lymphocytes on biopsy; Wood lamp accentuation does not exclude MF
Folliculotropic	Follicular papules, keratosis pilaris-like or acneiform lesions ± alopecia; often pruritic	Keratosis pilaris, acne vulgaris, primary follicular mucinosis, alopecia areata	Pruritus more frequent than in non-folliculotropic disease; requires deep biopsy showing folliculotropism ± mucin; trunk/limb follicular papules in a child
Classic patch/plaque	Thin scaly atrophic patches/plaques	Atopic dermatitis, psoriasis, nummular eczema, contact dermatitis, tinea corporis	Cytologic atypia (rather than architecture) discriminates from eczema; band-like epidermotropic infiltrate; dermoscopic features (linear/spermatozoa-like vessels, furrow scaling)
Poikilodermatous/rare variants	Poikiloderma; granulomatous slack skin; pagetoid reticulosis	Morphea, lichen sclerosus, genodermatoses with poikiloderma	Histopathologic confirmation essential; <5% of pediatric cases

**Table 3:** Recommendations for treatment of early-stage pediatric mycosis fungoides.

First-Line Therapy	Second-Line Therapy
Wait-and-see policy, mainly for stage IA patch	Skin-directed therapy:
Skin directed therapy:	PUVA-bath, UVA+NBUVB, UVA1, systemic PUVA
Potent/high potent topical corticosteroids, mainly for stage IA	Topical chlormethine (mechlorethamine), mainly for stage IA
NBUVB for patch/flat plaque	Topical bexarotene
UVA-based therapy for thicker plaques or FMF:	Systemic therapy:
PUVA-bath, UVA+NBUVB, or UVA1 are preferred over systemic PUVA	Retinoids (acitretin, isotretinoin) often with phototherapy
	Interferon alpha/Pegylated interferon-α 2a often with phototherapy
	Methotrexate

## Data Availability

No new data were created or analyzed in this study. Data sharing is not applicable to this article.
